# ROCK inhibitor enhances mitochondrial transfer via tunneling nanotubes in retinal pigment epithelium

**DOI:** 10.7150/thno.96508

**Published:** 2024-09-09

**Authors:** Jing Yuan, Fangxuan Chen, Dan Jiang, Zehua Xu, Hang Zhang, Zi-Bing Jin

**Affiliations:** 1 Beijing Institute of Ophthalmology, Beijing Tongren Eye Center, Beijing Tongren Hospital, Capital Medical University, Beijing, 100730, China.; 2 Clinical Pathology Diagnostic Center, Ningbo, Zhejiang, 315020, China.; 3 National Clinical Research Center for Ocular Diseases, Eye Hospital, Wenzhou Medical University, Wenzhou, 325027, China.

**Keywords:** ARPE19, cytoskeletal remodeling, light damage, mitochondrial transfer, nanotubes, Y-27632

## Abstract

**Rationale:** Tunnel nanotube (TNT)-mediated mitochondrial transport is crucial for the development and maintenance of multicellular organisms. Despite numerous studies highlighting the significance of this process in both physiological and pathological contexts, knowledge of the underlying mechanisms is still limited. This research focused on the role of the ROCK inhibitor Y-27632 in modulating TNT formation and mitochondrial transport in retinal pigment epithelial (RPE) cells.

**Methods:** Two types of ARPE19 cells (a retinal pigment epithelial cell line) with distinct mitochondrial fluorescently labeled, were co-cultured and treated with ROCK inhibitor Y-27632. The formation of nanotubes and transport of mitochondria were assessed through cytoskeletal staining and live cell imaging. Mitochondrial dysfunction was induced by light damage to establish a model, while mitochondrial function was evaluated through measurement of oxygen consumption rate. The effects of Y-27632 on cytoskeletal and mitochondrial dynamics were further elucidated through detailed analysis.

**Results:** Y-27632 treatment led to an increase in nanotube formation and enhanced mitochondrial transfer among ARPE19 cells, even following exposure to light-induced damage. Our analysis of cytoskeletal and mitochondrial distribution changes suggests that Y-27632 promotes nanotube-mediated mitochondrial transport by influencing cytoskeletal remodeling and mitochondrial movement.

**Conclusions**: These results suggest that Y-27632 has the ability to enhance mitochondrial transfer via tunneling nanotubes in retinal pigment epithelium, and similarly predict that ROCK inhibitor can fulfill its therapeutic potential through promoting mitochondrial transport in the retinal pigment epithelium in the future.

## Background

Material exchange between cells is crucial for the maintenance and development of multicellular organisms^1^-^4^, and intercellular mitochondrial transport serves as a prime example of such material exchange^1^, ^5^-^8^. Mitochondria, as vital organelles in eukaryotic cells, play a key role in providing cellular energy^9^, ^10^. Previous research has confirmed that mitochondrial transfer between cells is a common occurrence. Under physiological conditions, mitochondrial transfer is associated with tissue development and dynamic balance of energy^11^. In pathological conditions, mitochondrial dysfunction triggers more active mitochondrial transfer to replenish damaged cells with exogenous healthy mitochondria, aiming to restore cellular energy and facilitate disease regression^12^, ^13^. The transfer of mitochondria from donor to recipient cells appears to be a potential therapeutic modality for rescuing diseased cells^14^-^16^.

Among the mitochondrial transfer pathways reported to date, tunneling nanotubes (TNTs) are recognized as the principal cytoarchitecture for mitochondrial transportation^17^. The pioneering work by Rustom *et al.* in 2004 initially highlighted the role of TNTs in facilitating the transport of vesicles and organelles^18^. Subsequently, Spees *et al.* in 2006 demonstrated the transfer of functional mitochondria from mesenchymal stem cells (MSCs) to dysfunctional mammalian cells, thereby restoring aerobic respiration^19^. TNTs can be formed by the extension of filamentous pseudopods into neighboring cells or by separating two contacting cells^20^, ^21^. Structurally, TNTs are defined by their abundance of F-actin, suspension in the culture medium, and lack of attachment to the extracellular substrate^22^, ^23^. Functionally, TNTs exhibit the capacity to transport proteins, vesicles, organelles, RNA, and pathogens between nonadjacent cells^23^-^25^. Serving as a novel long-distance intercellular communication pathway, TNTs not only facilitate the exchange of intercellular cargoes and organelles but also contribute to intracellular homeostasis by balancing intercellular stress through material exchange^26^. However, the underlying molecular mechanisms governing TNT formation, particularly the endogenous mechanisms, remain elusive.

Currently known methods that can regulate TNT-mediated mitochondrial transport include two aspects: one is increasing the chance of mitochondrial transport occurring by increasing the demand for mitochondrial energy synthesis of recipient cell^27^-^30^; another is increasing the number of mitochondria transport by enhancing the expression of relevant transporter proteins in the donor cell^31^. The cytoskeleton is a major component of TNT, and mitochondrial transport within the cell is to some extent dependent on the cytoskeleton. Here, we propose a new conjecture that TNT is essentially a specialized filamentous protrusion, so could TNT formation be increased by altering the arrangement of the cytoskeleton thereby further increasing the chances of mitochondrial transfer between cells?

Rho GTPases play a crucial role in regulating cytoskeleton dynamics, with key members including RhoA, Rac1, and CDC42. The downstream protein of RhoA, ROCK, can be inhibited by Y-27632^32^, ^33^. Our previous research has demonstrated the transfer of mitochondria between mesenchymal stem cells (MSCs) and different types of ocular cells through tunneling nanotubes based on F-actin^1^. Additionally, studies by Robin Ali have shown that mammalian photoreceptor neurons can form open nanotube-like protrusions and mediate mitochondrial transfer^34^. In this study, we focused on the retinal pigment epithelium (RPE) to investigate the effect of the ROCK inhibitor Y-27632 on the pattern of mitochondrial transfer between homologous cells, as well as exploring the potential benefits of enhanced mitochondrial transport on self-repair in cells with dysfunctional mitochondria. We look forward to bringing new insights into intercellular mitochondrial transfer mechanisms.

## Results

### Y-27632 enhanced intercellular nanotube formation and mitochondrial transfer

Previous studies have shown that actin polymerization is essential for the formation of TNT, with the RhoGTPase family, particularly ROCK, playing a regulatory role in the actin cytoskeleton^35^, ^36^. Y-27632, a specific inhibitor of the ROCK pathway, has been reported to promote axon extension in nerve injury^37^. Therefore, we investigated the potential of Y-27632 in modulating TNT formation.

**Figure 1:**
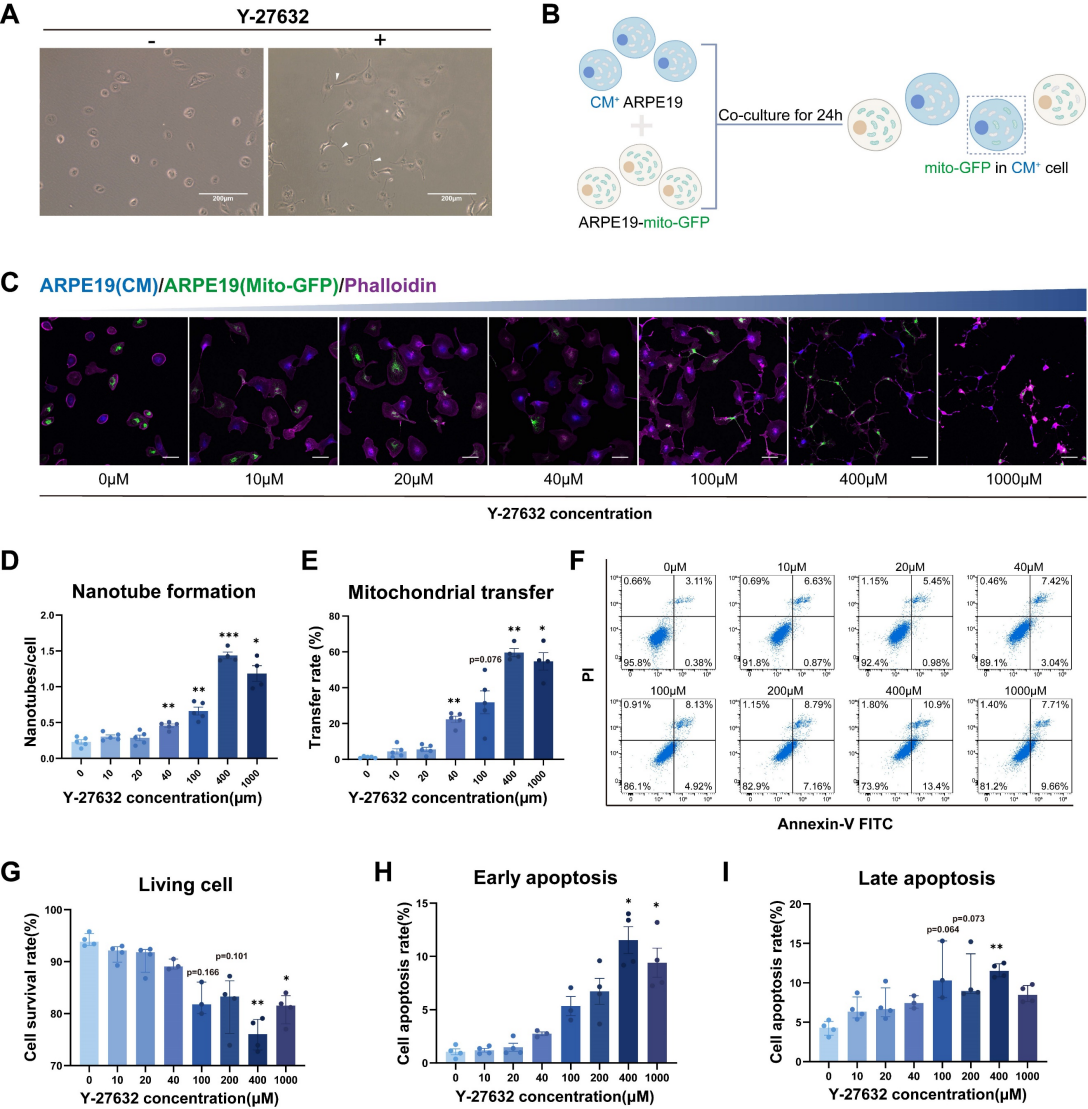
** Y-27632-induced intercellular nanotube formation and mitochondrial transfer are dose-dependent.** (A) Images captured under bright-field microscopy of nanotubes in ARPE19 cells treated with the ROCK inhibitor Y-27632 (10µM) for 24 hours are shown, with nanotubes indicated by white triangles. Scale bar: 200µm. (B) A diagram illustrating the co-culture program. ARPE19 cells expressing mito-GFP (green) were co-cultured as donor cells in direct contact with ARPE19 cells stained with cytoplasmic membrane (CM, blue) as recipient cells at a ratio of 1:1 for 24 hours. Mito-GFP in CM^+^ cells marked mitochondrial transfer. (C) ARPE19-mito-GFP (green) and ARPE19 cells (CM, blue) were co-cultured and treated with varying concentrations of Y-27632 for 24 hours to induce nanotube formation. Nanotube formation and mitochondrial transfer were imaged by confocal microscopy. Scale bar: 50µm. (D, E) The number of nanotubes each cell formed and mitochondrial transfer rate were quantified. n=5, one-way ANOVA with Dunnett's T3 multiple comparisons post hoc tests, mean±SEM; *p<0.05, **p<0.01, ***p<0.001. (F-I) Representative flow cytometry images of Annexin V-FITC/PI analysis for ARPE19 cells with treatment of varying concentrations of Y-27632 for 24 hours (F). The percentage of living cells (Annexin V-FITC^-^/PI^-^) (G), early apoptotic cells (Annexin V-FITC^+^/PI^-^) (H) and late apoptotic cells (Annexin V-FITC^+^/PI^+^) (I) were quantified. n=4, Kruskal-Wallis test, median ± interquartile range; *p<0.05, **p<0.01.

Here, we investigated the potential of Y-27632 treated ARPE19 cells to form intercellular nanotubes, and the exchange of organelles, specifically mitochondria, between ARPE19 cells through this mechanism. Thin, membranous structures connecting two cells were observed following the addition of 10µM Y-27632 to the culture medium (Figure 1A). Intercellular transfer of mitochondria was assessed using mito-GFP labeled ARPE19 cells as donor cells and ARPE19 cells stained with cell trace violet (cytoplasmic membrane, CM) as recipient cells. The two types of cells were co-cultured at a 1:1 ratio (Figure 1B). Nanotube formation and mitochondrial transport between ARPE19 cells were visualized using confocal microscopy followed by treatment with varying concentrations of Y-27632 for 24 hours (Figure 1C). Phalloidin staining demonstrated a significant increase in Y-27632-induced nanotube formation with escalating concentrations of Y-27632, especially when it was increased to 40µM (Figure 1D). Moreover, a notable enhancement in mitochondrial transfer rate was observed with increasing Y-27632 concentration, suggesting a potential role for Y-27632 in facilitating nanotube-mediated mitochondrial transfer between ARPE19 cells (Figure 1E). In order to confirm the role of ROCK inhibitors in promoting nanotube formation and mitochondrial transfer, we further investigated the effects of the ROCK inhibitors Y-39983 and GSK269962A on nanotube formation and mitochondrial transfer in ARPE19 cells. The findings revealed that treatment with 10µM of Y-39983 and 10µM of GSK269962A also resulted in a significant enhancement of both nanotube formation and mitochondrial transfer in ARPE19 cells (Figure S1A, B and C). Subsequently, the impact of escalating Y-27632 concentrations on ARPE19 cells was evaluated. Quantitative analysis of Annexin V and PI assays revealed a decrease in cell viability and an increase in apoptosis rates in ARPE19 cells treated with concentrations exceeding 40µM (Figure 1F, G, H, and I). Therefore, 40µM of Y-27632 treatment was selected for inducing nanotube formation and mitochondrial transfer in subsequent experiments.

### Characterization of Y-27632-induced intercellular nanotubes

**Figure 2:**
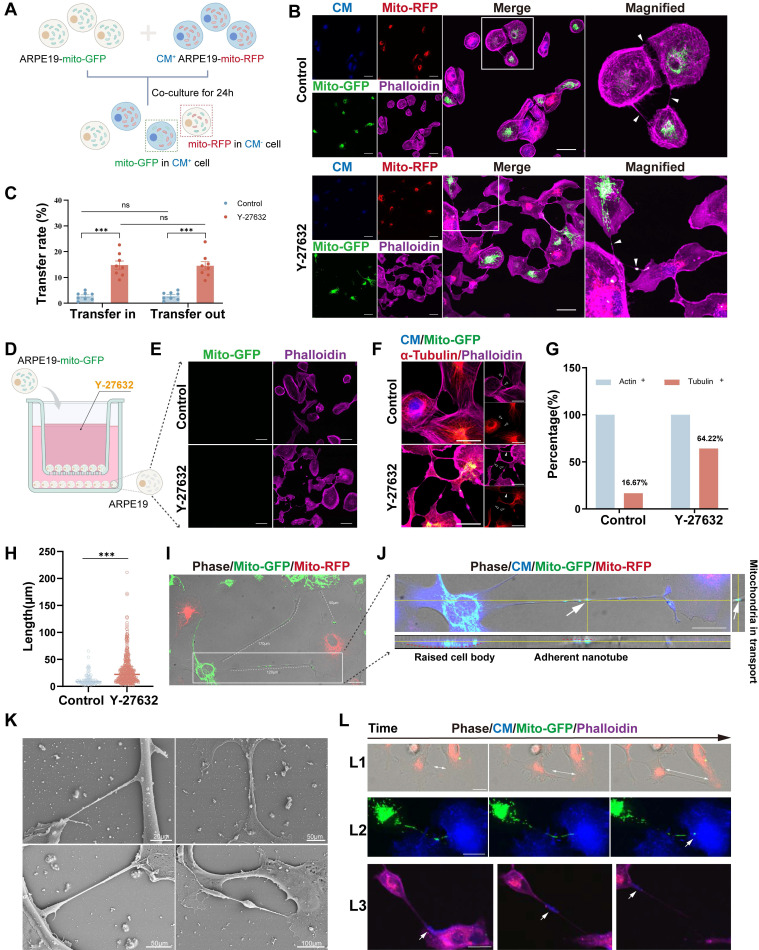
** Characterization of Y-27632-induced nanotubes.** (A) A diagram illustrating the co-culture experiments. ARPE19-mito-GFP cells (green) and ARPE19-mito-RFP cells (red, CM, blue) co-culture at 1:1 ratio for 24 hours. Mito-GFP in CM^+^ cells marked mitochondrial transfer from ARPE19-mito-GFP cells to ARPE19-mito-RFP cells while mito-RFP in CM^-^ cells marked mitochondrial transfer from ARPE19-mito-RFP cells to ARPE19-mito-GFP cells. (B) ARPE19-mito-RFP cells (blue) were co-cultured with ARPE19-mito-GFP cells (green) for 24 hours. All cells were stained with phalloidin (magenta) followed by fluorescent confocal microscopy. White triangles indicate nanotubes with or without mitochondria. Scale bar: 50µm. (C) Mitochondrial transfer rate including transfer in and transfer out of ARPE19 cells were quantified in control and Y-27632 groups. n=8, two-way ANOVA test, mean±SEM; ns, not significant, ***p<0.001. (D) A diagram showing that ARPE19-mito-GFP cells (green) were co-cultured as donor cells with ARPE19 as recipient cells at 1:1 ratio, with the two types of cells separated by filters in transwell. (E) Representative images of ARPE19 cells (phalloidin, magenta) in the bottom of a transwell plate. Scale bar:50 µm. (F) F-actin (phalloidin, magenta) and microtubules (α-tubulin, red) of nanotubes were detected in ARPE19 cells in control and Y-27632 groups. White solid triangles indicate nanotubes containing both F-actin and microtubules, while hollow triangles indicate nanotubes containing F-actin, lacking microtubules. Scale bar: 50 µm. (G) The percentage of nanotubes containing F-actin (actin+) and microtubules (tubulin+) were quantified.n (control)=42, n (Y-27632)=218. (H) Quantification of the length of intercellular nanotubes in control and Y-27632 groups. n (control)=91, n (Y-27632)=453. Mann-Whitney test, median ± interquartile range; ***p<0.001. (I) Intercellular nanotubes of different lengths are formed between ARPE19-mito-GFP and ARPE19-mito-RFP cells following treatment with Y-27632. The white dotted line marks the nanotube with their length measured. Scale bar: 25 µm. (J) Nanotubes shown by CM staining (blue) were adherent to the substrate (X-Z axes). Scale bar: 25 µm. (K) Representative images of scanning electron microscope (SEM) show different kinds of Y-27632-induced intercellular nanotubes. (L) Time-lapse microscopic images taken from Movie 1, 2 and 3 showing that intercellular nanotube formation between ARPE19-mito-GFP (green) and ARPE19-mito-RFP cells (red) (white arrows indicate the direction of cell movement) (L1), mitochondrial transfer from ARPE19-mito-GFP cells (green) to ARPE19 cells (blue) (L2), and some other cargoes (blue) are transported between ARPE19 cells (magenta) (L3). Scale bar: 25 µm.

To further investigate mitochondrial exchange, stable ARPE19-mito-GFP and ARPE19-mito-RFP cell lines were generated, wherein mitochondria were labeled with mito-GFP and mito-RFP, respectively (Figure S2A and B). Co-culturing ARPE19-mito-GFP cells with ARPE19-mito-RFP cells stained with cytoplasmic membrane (CM) at a 1:1 ratio for 24 hours revealed the presence of GFP-labeled mitochondria derived from donor cells in CM^+^ recipient cells (Figure 2A and B). Additionally, Y-27632 treatment was found to enhance nanotube formation and mitochondrial transfer (Figure S3A and B). The capacity of cells to both receive mitochondria from other cells (transfer in) and transfer their mitochondria to other cells (transfer out) was quantified following Y-27632 treatment. Cells in a normal state have no significant difference in the ability of mitochondria to transfer in and out, whether or not enhanced by using Y-27632 (Figure 2C). To investigate the role of Y-27632-induced nanotube in mitochondrial transfer, we utilized a co-culture test that was previously documented^1^. In this experimental setup, ARPE19 cells labeled with mito-GFP were designated as the donor cells, co-culturing with unlabeled recipient ARPE19 cells in a transwell with a 0.4µm pore size filter separating the two cell populations (Figure 2D). Recipient cells that received mitochondria from donors were examined using confocal microscopy after being co-cultured for 24 hours. Cell-cell contact was necessary for the observed transfer of mitochondria, as the transfer was nearly undetectable when recipient and donor cells were cultured separately in a transwell with a filter barrier (Figure 2E). F-actin typically serves as a crucial constituent of tunneling nanotubes (TNTs), with the presence of tubulin also noted in specific cell types exhibiting thicker TNTs. Some studies have indicated that TNTs between ARPE19 cells exclusively consist of F-actin^38^. Consequently, we investigated the composition of Y-27632-induced nanotubes (Figure 2F). Our findings revealed that the majority (83.33%) of nanotubes between untreated ARPE19 cells comprised solely F-actin. However, approximately 64.22% of Y-27632-induced nanotubes contained both F-actin and tubulin (Figure 2G). It is plausible that TNTs containing microtubules may facilitate mitochondrial transport due to their larger diameter.

Intercellular nanotubes exhibit a wide range of lengths, varying from a few to over a hundred micrometers, and may display curvature and branching, as evidenced in ARPE19 cells (Figure 2I, Figure S2C). The length of nanotubes in both control and Y-27632 treated groups was quantified. Y-27632-induced nanotubes demonstrated a significantly greater average length (30.00±27.28µm) compared to nanotubes in the control group (12.48±11.04µm) (Figure 2H). Given the characteristic feature of TNTs being non-adherent to the extracellular substrate, we conducted z-axis imaging to examine these membranous structures. Staining of the cytoplasmic membrane (CM) revealed that these structures were indeed adherent to the extracellular substrate (Figure 2J). The Y-27632-induced nanotubes appear distinct from conventional TNTs, hence termed as Y-NTs. Scanning electron microscope (SEM) images depict various types of Y-NTs (Figure 2K).

To validate the functionality of Y-NTs in ARPE19 cells for transporting cargoes like mitochondria between interconnected cells, we conducted a live cell imaging assay using ARPE19-mito-GFP cells and CM^+^ ARPE19 cells. Through time-lapse confocal microscopy (see Movie 1, 2, and 3), we observed the formation of Y-NTs (Figure 2L1), the entry of mitochondria into recipient cells (Figure 2L2), and the movement of mitochondria along Y-NTs (Figure 2L3). These findings provide evidence that Y-NTs facilitate mitochondrial transfer between ARPE19 cells.

### Cytoskeletal inhibition eliminates Y-NTs-mediated mitochondrial transfer

**Figure 3:**
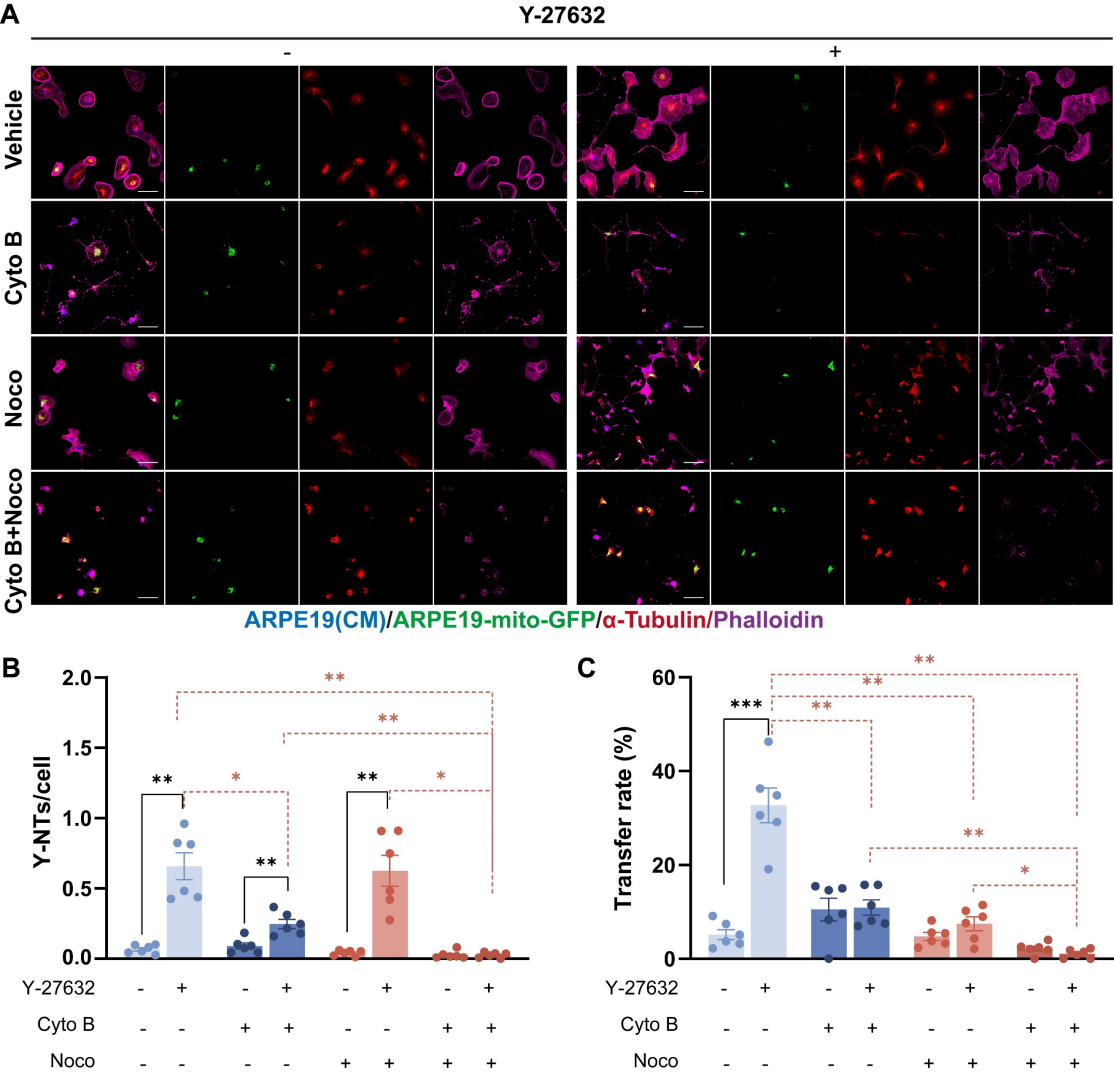
** Cytoskeletal inhibition eliminates Y-NTs mediated mitochondrial transfer.** (A) ARPE19-mito-GFP cells (green) co-cultured with ARPE19 cells (blue) in control and Y-27632 groups were treated with Cytochalasin B (CytoB) (10µM, 24h), Nocodazole (Noco) (50µM, 24h) and stained with phalloidin (magenta) and anti-α-tubulin antibody (red). Scale bar: 50µm. (B, C) Intercellular nanotube formation and mitochondrial transfer rate were quantified in each group. n=6, Comparison of the effects of different inhibitor treatments on the control and Y-27632 groups was performed using t-tests, and comparison of the effects of different inhibitor treatments within the control or Y-27632 groups was performed using one-way ANOVA. mean±SEM; *p<0.05, **p<0.01, ***p<0.001. Here, we have unified the results into a single graph for ease of presentation.

The interaction between mitochondria and the cytoskeleton is essential for the proper functioning of mitochondria^39^. Previous studies have indicated that the movement of mitochondria over long distances is facilitated by microtubules, whereas short-term movement is mediated by actin filaments^40^. To investigate whether the increase of mitochondrial transfer in Y-27632 treated cells depends on the actin filaments or microtubules, we utilized Cytochalasin B (Cyto B) and Nocodazole (Noco), which are known to depolymerize F-actin and microtubules, respectively. After treating with either Cyto B or Noco, in addition to vehicle control (DMSO), the formation of Y-NTs and mitochondrial transfer were assessed using confocal microscopy (Figure 3A). In Y-27632 treated cells, depolymerization of F-actin inhibited the formation of nanotubes and the transfer of mitochondria. Similarly, treatment with Nocodazole reduced mitochondrial transport, yet did not completely abolish the stimulatory effect of Y-27632 on nanotube formation (Figure 3B and C). Our findings demonstrate that the formation of Y-NTs is predominantly reliant on actin filaments, while the transport of mitochondria may depend on microtubules for Y-27632-induced long-distance movement.

### Y-NTs mediated mitochondrial transfer rescues light-damaged ARPE19 cells

Functional mitochondrial transfer has the potential to rescue cells with mitochondrial dysfunction, which always requires MSC involvement^27^-^30^. Our study aimed to investigate the impact of Y-27632 on enhancing the self-repair capacity of ARPE19 cells through modulation of mitochondrial transport via Y-NTs. To assess the efficacy of this modulation, we conducted experiments using a co-culture assay and subjected the cells to light damage (LD) to evaluate their survival rates.

**Figure 4:**
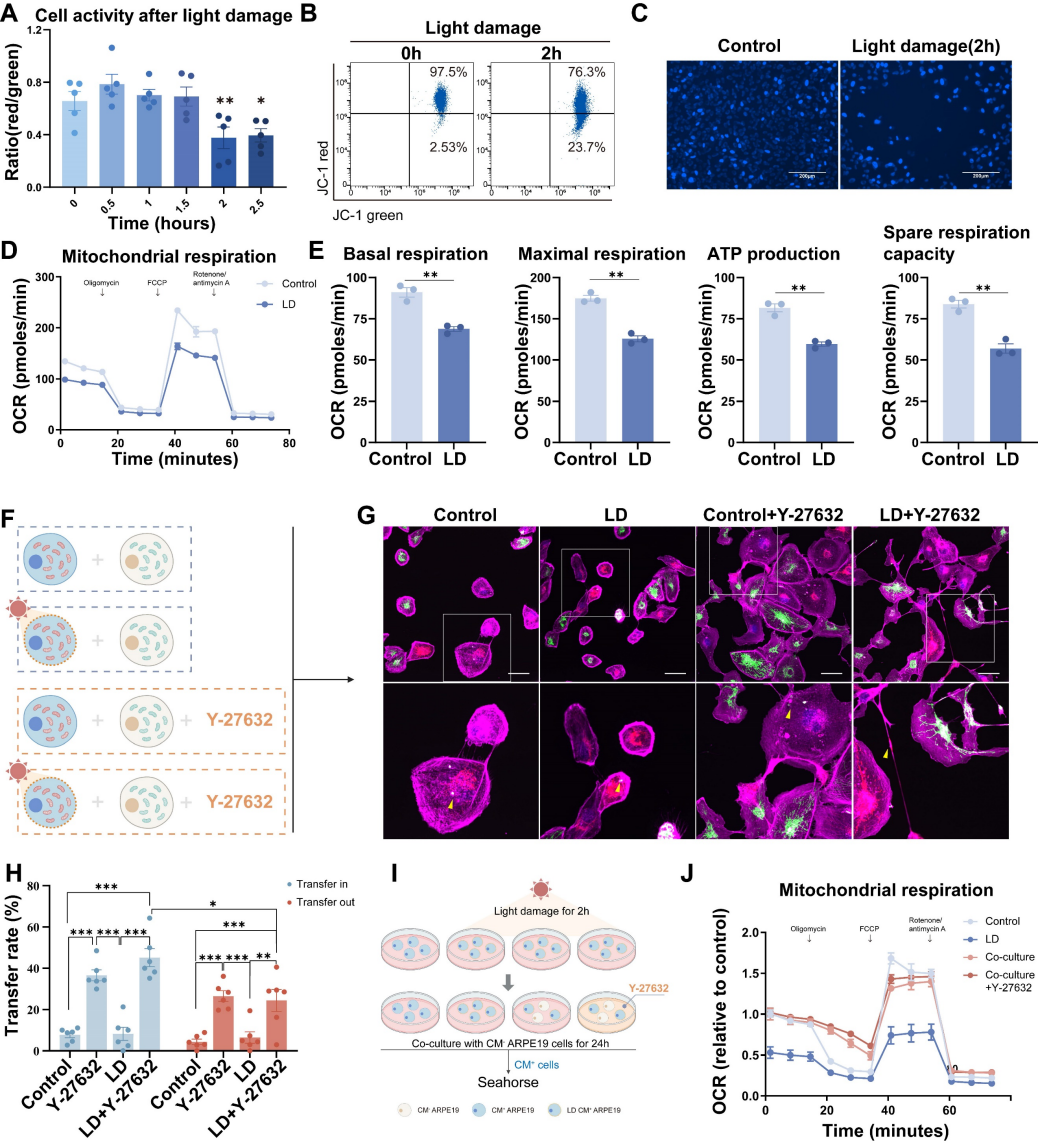
** Y-NTs mediated mitochondrial transfer rescues light-damaged ARPE19 cells.** (A) Cell activity shown as the ratio of red/green by JC-1 test with light exposure of different durations. n=5, one-way ANOVA test, mean±SEM; *p<0.05. (B) Representative flow cytometry plot of the result of the JC-1 test for ARPE19 cells after light damage for 0 hours and 2 hours. (C) Representative images of Hoechst 33342 staining (blue) of ARPE19 cells after light damage for 0 hours and 2 hours. Scale bar: 200µm. (D) Extracellular oxygen consumption rate (OCR) analysis of ARPE19 cells after 2 hours of light damage by seahorse. (E) Quantification of oxygen consumption rate (OCR) in mitochondrial basal respiration, maximal respiration, ATP production, and spare respiratory capacity. n=3, t-test, mean±SEM; ns, not significant,**p<0.01. (F) A diagram showing that CM^+^ ARPE19-mito-RFP cells with or without light damage were co-cultured with healthy ARPE19-mito GFP cells at a 1:1 ratio in the absence or presence of Y-27632. (G) Representative images of co-cultured light-damaged ARPE19-mito-RFP cells (blue) with healthy ARPE19-mito GFP cells (green), all cells were stained with phalloidin (magenta). Enlarged box area displaying the donor-derived mitochondria (green) in recipient CM^+^ cells (blue). Yellow triangles indicate mitochondrial transfer. Scale bar: 50µm. (H) Mitochondrial transfer rates including transfer in and transfer out of light-damaged ARPE19 cells were quantified in different groups. Scale bar: 50µm. n=6, Comparison of the effects of different treatments on the mitochondrial transfer rate between ARPE19 cells was performed by one-way ANOVA, and t-test was used to compare the mitochondrial transfer rate into and out of ARPE19 cells under the same treatment., mean±SEM; ns,not significant,*p<0.05,**p<0.01, ***p<0.001. Here, we have unified the results into a single graph for ease of presentation. (I) A diagram showing that CM^+^ ARPE19 cells with or without light damage were further cultured or co-cultured with healthy ARPE19 cells at a 1:1 ratio in the absence or presence of Y-27632 and analyzed by seahorse. (J) Extracellular oxygen consumption rate (OCR) analysis of ARPE19 cells in different groups normalized to control.

To validate our findings, we developed a light damage model of mitochondrial dysfunction^41^. ARPE19 cells were initially exposed to varying durations of blue light (19klux, 330-340nm) to induce light damage. Flow cytometry analysis was used to quantify mitochondrial dysfunction by labeling JC-1 in different experimental groups (Figure 4A). A 2-hour exposure duration was selected for subsequent experiments, during which a 23.7% decrease in mitochondrial membrane potential was observed (Figure 4B). Hoechst 33342 staining also revealed light-induced apoptosis (Figure 4C). Additionally, light-induced mitochondrial dysfunction was assessed using the XF cell mito stress test (Figure 4D), which showed significant reductions in both basal and maximal respiratory capacity, ATP production and spare respiratory capacity following light damage (Figure 4E).

Subsequently, alterations in mitochondrial transfer patterns were evaluated. The experiments were conducted following the programs in Figure 4F. Co-cultures were established between CM^+^ ARPE19-mito-RFP cells, with or without light-induced damage, and healthy ARPE19-mito-GFP cells at a 1:1 ratio, in the absence or presence of Y-27632 (Figure 4F). The transfer of mito-GFP labeled mitochondria from ARPE19-mito-GFP to ARPE19-mito-RFP cells was visualized as punctate green or yellow regions in CM^+^ light-damaged cells (Figure 4G). Light damage did not augment mitochondrial transfer in ARPE19 cells; however, Y-27632 treatment potentiated mitochondrial transfer under both normal and light damage conditions. Furthermore, light-damaged ARPE19 cells exhibited a significantly higher propensity for mitochondrial transfer inward compared to outward transfer (Figure 4H). These findings indicate that Y-27632 modulates mitochondrial transfer patterns in cells with mitochondrial dysfunction.

Following light-induced damage, CM^+^ ARPE19 cells were subsequently cultured or co-cultured with healthy ARPE19 cells at a 1:1 ratio in the presence or absence of Y-27632. After a 24-hour incubation period, mitochondrial function was assessed using seahorse analysis (Figure 4I). Co-cultivation with healthy cells resulted in a significant reversal of the energy metabolism impairment observed in light-damaged cells compared to monocultured cells (Figure 4J, Figure S4A), as evidenced by increased basal respiration, maximal respiration, and spare respiration capacity (Figure S4B). Unfortunately, treatment with Y-27632 did not yield a discernible difference in co-cultured cells compared to untreated co-cultured cells.

### Y-27632 regulates cytoskeleton remodeling

**Figure 5:**
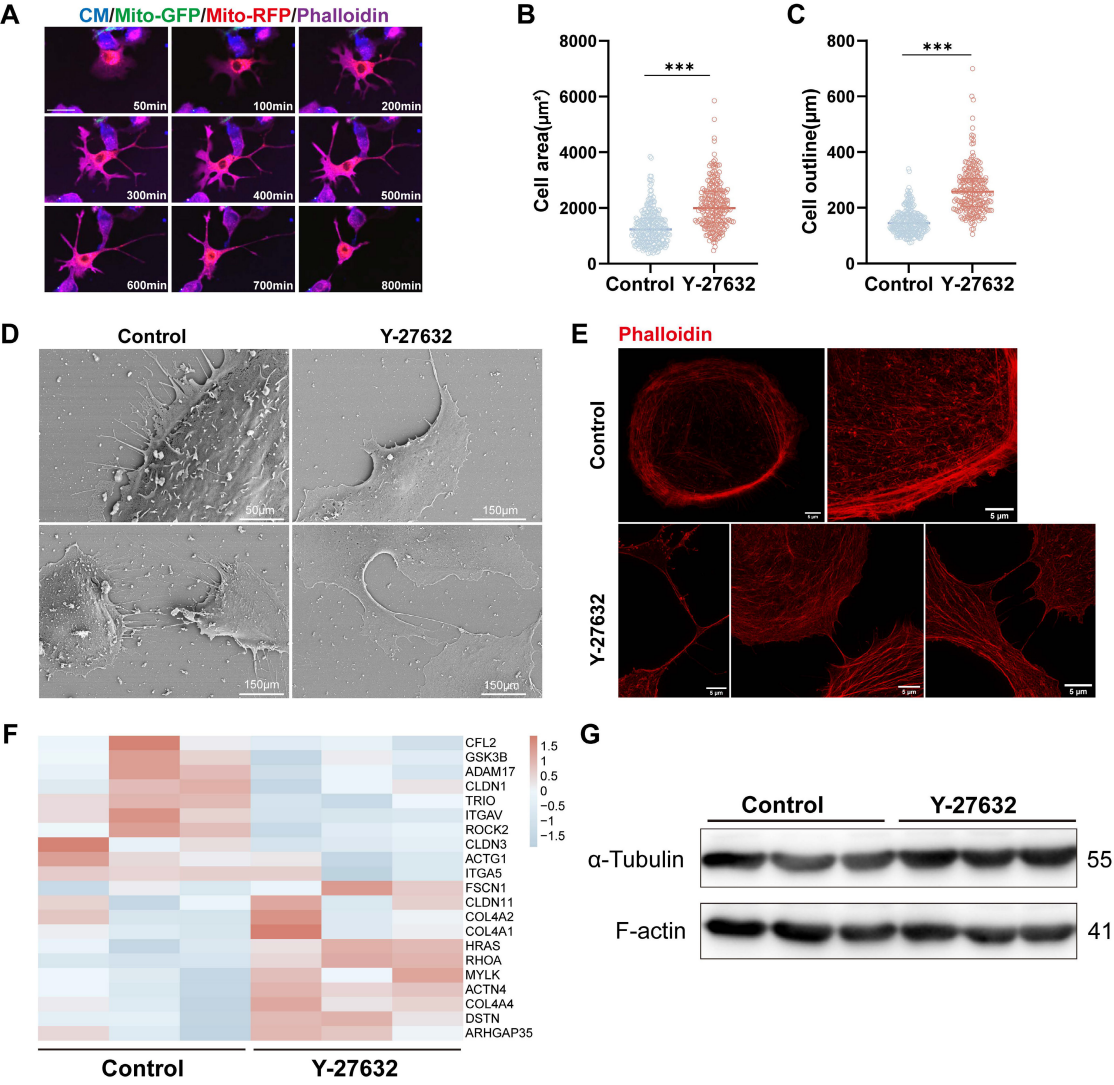
** Y-27632 regulates cytoskeleton remodeling.** (A) Time-lapse microscopic images taken from Movie 2 showing the morphological change in an ARPE19 cell (phalloidin, magenta) treated with 40µM Y-27632. Scale bar: 50µm. (B-C) Quantitative statistics of cell area (B) and outline length (C) of ARPE19 cells in control and Y-27632 groups. n (control)=256, n (Y-27632)=228.Mann-Whitney test, median ± interquartile range; ***p<0.001. (D) Representative images of scanning electron microscope (SEM) show morphological differences between ARPE19 cells in control and Y-27632 groups. (E) Super-resolution confocal imaging reveals changes in the alignment of microfilaments at the edge of the contour of Y-27632-treated ARPE19 cells. Scale bar: 5µm. (F) Heatmap showing differences in the expression of some cytoskeletal molecules in ARPE19 cells after Y-27632 treatment, but not statistically significant. (G) Microfilament (F-actin) and microtubule (α-tubulin) protein levels were detected in control and Y-27632 groups by western blotting.

Previous research has shown that the Rho GTPase family plays a role in regulating the cytoskeleton, specifically F-actin. It has been documented that F-actin dynamics are crucial for the formation of TNTs^35^, ^36^, ^42^. Therefore, we investigated the impact of Y-27632 on F-actin polymerization in ARPE19 cells. Live cell imaging (see Movie 2) revealed significant changes in cytoskeletal organization and cell movement upon Y-27632 treatment compared to untreated cells, with ARPE19 cells extending axon-like structures to establish connections with neighboring cells (Figure 5A). Furthermore, the average cell attachment area increased from 1307±619.4µm² to 2089±827.3µm², and the cell outline length expanded from 150.5±43.74µm to 264.0±83.74µm following Y-27632 treatment (Figure 5B and C). To examine the intricate structure of cell morphology, the cells were analyzed using scanning electron microscopy and super-resolution confocal microscopy. Normal ARPE19 cells exhibited prominently raised cytosol and tightly organized cytosolic actin filaments. Upon treatment with Y-27632, the cells displayed a more malleable and pliable nature, facilitating the formation of various structures such as filamentous pseudopods, platelike feet, and inter-cellular connections. Additionally, the distribution of microfilaments throughout the entire cell was more uniform and consistent (Figure 5D and E). These findings suggest an increased likelihood of intercellular nanotube formation compared to control cells, which exhibited minimal changes in cell morphology (see Movie 4).

Subsequent investigation delved into the impact of Y-27632 on cytoskeletal alterations at the molecular protein level. RNA sequencing analysis unveiled a tendency towards differential expression in certain cytoskeletal molecules, albeit lacking statistical significance (Figure 5F). Protein expression of F-actin and tubulin similarly exhibited no significant alterations (Figure 5G). These findings suggest that treatment with 40µM Y-27632 induced cytoskeletal reorganization without eliciting notable changes in the expression of key skeletal proteins.

### Y-27632 promotes mitochondrial movement in response to cytoskeletal changes

**Figure 6:**
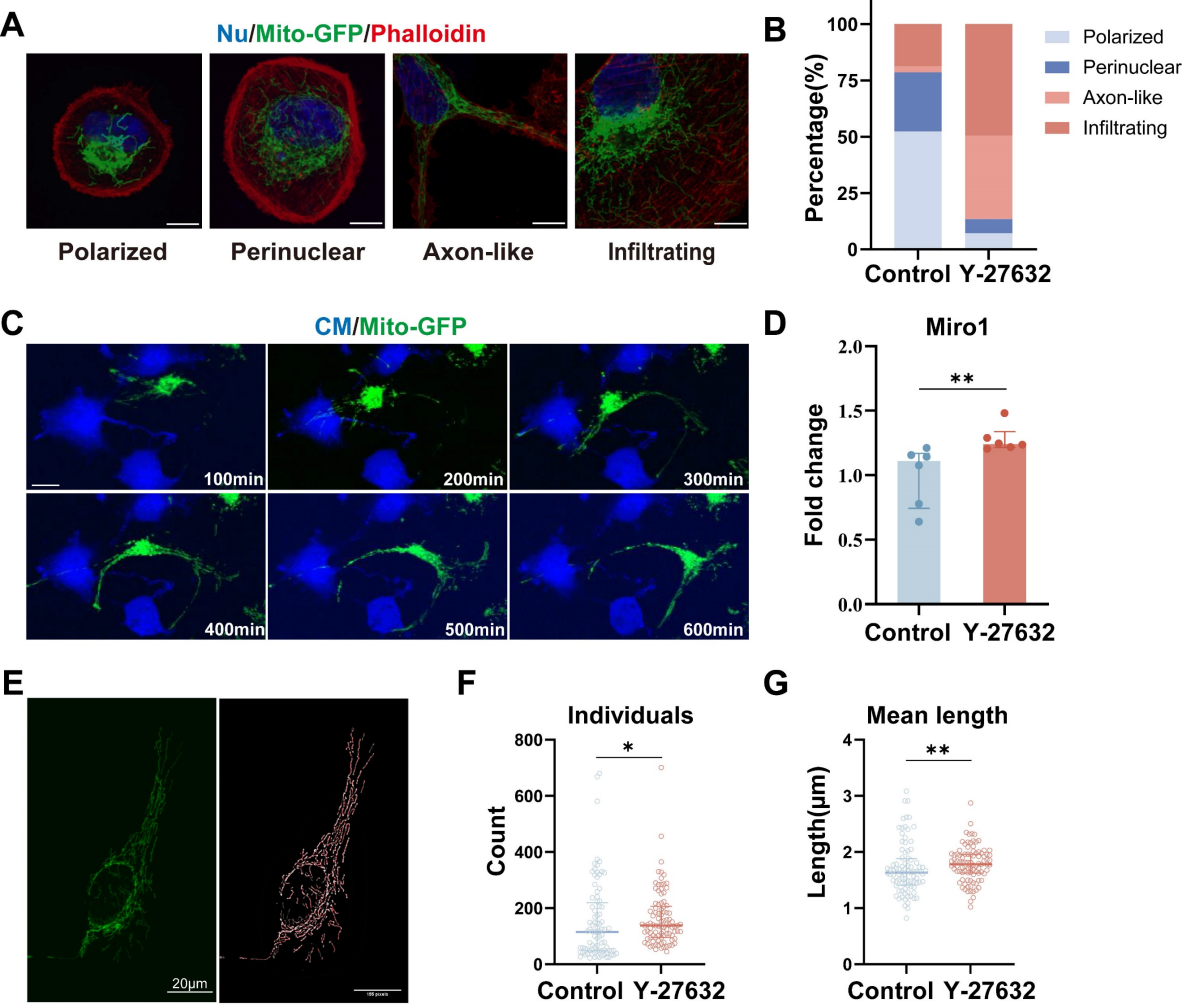
** Y-27632 promotes mitochondrial movement in response to cytoskeletal changes.** (A) Four mitochondrial distribution patterns identified by confocal microscopy. ARPE19-mito-GFP cells (green) were stained with phalloidin (red) and DAPI (blue). Images displaying different distribution patterns of mitochondrial (polarized, perinuclear, axon-like, and infiltrating). Scale bar: 25 µm. (B) Quantifying the proportion of cells exhibiting various patterns of mitochondrial distribution (polarized, perinuclear, axon-like, and infiltrating). n (control)=107, n (Y-27632) =97. (C) Time-lapse microscopic images taken from Movie 3 showing the movement of mitochondria (green) in an ARPE19 cell treated with 40µM Y-27632. Scale bar: 25 µm. (D) Quantitative RT-PCR was performed to detect miro1 mRNA levels. n=6, Mann-Whitney test, median ± interquartile range; **p<0.01. (E) Intact mitochondria of an ARPE19-mito-GFP cell (green, left) and its mitochondrial network skeleton extracted by image J software (right). Scale bar: 20µm. (F-G) The number and average length of individual mitochondria in control and Y-27632 groups were quantified. n (control)=88, n (Y-27632)=95. Mann-Whitney test, median ± interquartile range; *p<0.05, **p<0.01.

Subcellular distribution of mitochondria was assessed to determine the impact of Y-27632. Mitochondria were classified based on established subcellular distribution patterns^43^, and quantitative analysis was conducted in control and Y-27632 groups. Similar to the published study^43^, we identified three categories of mitochondrial arrangement: polarized (grouped around one side of the nucleus), perinuclear (spread around the nucleus), and infiltrating (entering the cortical cytoskeleton). Additionally, a novel mitochondrial distribution pattern termed axon-like (forming bundles resembling axons) was observed in the Y-27632 group that could not be categorized into the three previously mentioned (Figure 6A). Control cells predominantly exhibited a "polarized" and "perinuclear" pattern, while Y-27632 treated cells displayed elongated mitochondria with an "infiltrating" and "axon-like" pattern. The percentage of "polarized" and "perinuclear" mitochondria decreased from 52.34% and 26.17% to 7.22% and 6.19%, respectively, while "infiltrating" and "axon-like" mitochondria increased from 18.69% and 2.80% to 49.48% and 37.11%, respectively (Figure 6B).

In the live cell imaging assay conducted (see Movie 3), it was observed that mitochondria exhibited a tendency to cluster around the nucleus and displayed minimal movement in control cells. Conversely, in cells treated with Y-27632, a significant portion of mitochondria demonstrated heightened mobility and penetrated into the cortical cytoskeleton, aligning with cytoskeletal dynamics (Figure 6C, Movie 2). The movement of mitochondria was found to be mediated by the miro1 protein, which governs mitochondrial transport along microtubules by facilitating the connection between mitochondria and motor proteins^39^, ^44^. Notably, treatment with Y-27632 was shown to impact mitochondrial dynamics by upregulating the expression of miro1 (Figure 6D, Figure S5A, and B). As a member of the GTPase family and localized on the outer mitochondrial membrane, miro1 plays a pivotal role in regulating mitochondrial transport and distribution^45^. Recent studies have highlighted the ability of the miro1 protein to coordinate mitochondrial movement and positioning through interactions with actin and microtubules^46^.

To further analyze the impact of Y-27632 on mitochondria, we conducted mitochondrial network structure analysis using ImageJ software (Figure 6E). Quantitative assessment revealed an increased number of individual mitochondria in cells treated with Y-27632 (Figure 6F). Moreover, the average length of individual mitochondria in Y-27632-treated cells was significantly greater compared to untreated cells (Figure 6G). The network number and size of mitochondria showed no significant differences with Y-27632 treatment (Figure S4D and E). These findings suggest that Y-27632 maintains mitochondria in a metabolically active and readily transportable state, facilitating their infiltration into the cortical cytoskeleton.

## Discussion

Over the nearly two decades since their discovery, tunneling nanotubes (TNTs) have been acknowledged as an additional mechanism for intercellular communication, facilitating the exchange of various cellular cargo among cells, and they have shown significant potential in various diseases and pathological processes 21, 23. Despite the identification of TNTs and TNT-like structures in diverse cell types cultured *in vitro*, such as immune cells, kidney cells, epithelial cells, tumor cells, neurons, astrocytes, and retinal cells, there is a substantial gap in understanding the molecular mechanisms governing the formation and functionality of these enigmatic structures^47^. The outward polymerization, targeted regulation, and local deformation of the plasma membrane of F-actin all promote the outward extension of TNT^23^.

In this study, we observed that the ROCK inhibitor Y-27632 stimulates an increase in nanotube-mediated mitochondrial transfer among retinal pigment epithelial cells. In addition to Y-27632, the ROCK inhibitors Y-39983 and GSK269962A also elicited similar effects on ARPE19 cells. Several studies in other cells on the promotion of mitochondrial transfer by ROCK inhibitors similarly support the present study^42^, ^48^, ^49^. However, our findings first indicate that Y-27632 induces alterations in cell morphology by regulating the organization of cytoskeletal elements, specifically actin filaments and microtubules, leading to the formation of protrusion-like structures that extend into neighboring cells, thereby promoting nanotube formation. These nanotubes, enriched with microtubule structures, facilitate frequent mitochondrial transport. Furthermore, Y-27632 promotes the intracellular movement of mitochondria from perinuclear clusters to a dispersed distribution within the cellular cytoskeleton, accompanied by the extension of cellular protrusions, which in turn enhances transcellular mitochondrial transport. Disruption of actin filaments and microtubules abolishes the facilitating effect of Y-27632. Notably, light-damaged recipient ARPE19 cells treated with Y-27632 exhibit increased mitochondrial respiration following the uptake of more mitochondria from healthy ARPE19 cells. These results suggest that the promotion of mitochondrial transfer between homologous cells can be achieved by pharmacological intervention to enhance cellular self-repair mechanisms, rather than relying on exogenous mitochondrial donor cells.

Our findings suggest that mitochondrial intercellular transfer is a natural physiological process in retinal pigment epithelium (RPE) cells, and treatment with Y-27632 (40µM) promotes an increase in the formation of RPE intercellular nanotubes (Y-NTs) and enhances the rate of mitochondrial transfer. Compared to regular TNTs, Y-NTs exhibit longer average lengths, leading to a higher presence of microtubules alongside F-actin and attachment to extracellular substrates. These characteristics contribute to a more robust structure, increased resistance to mechanical damage, and facilitate longer-distance material transport. The development of Y-NTs relies heavily on actin filaments and microtubules, with mitochondrial transport being predominantly microtubule-dependent. Inhibition of actin filament and microtubule aggregation abolishes the Y-27632-induced increase in nanotubules and mitochondrial transfer. Disruption of actin filaments alone results in a reduction in Y-NT formation and mitochondrial transfer, while disruption of microtubules alone decreases mitochondrial transfer without affecting the number of TNTs. These findings suggest that Y-NT formation primarily hinges on actin filaments, whereas Y-27632-induced mitochondrial transfer is predominantly reliant on microtubules.

Mitochondria serve as the primary cargo transported through tunneling nanotubes (TNTs), with TNT-mediated mitochondrial transfer potentially affecting cell survival and adaptation^47^. For instance, the transfer of mitochondria via TNTs in healthy cells has been shown to rescue cell apoptosis triggered by UV exposure^50^. In this investigation, we assessed the efficacy of mitochondrial transfer in both inbound and outbound directions. In Y-27632-treated light-damaged retinal pigment epithelial (RPE) cells, the capacity for inbound mitochondrial transfer was found to be more robust than outbound transfer. Our findings also indicate that Y-27632 treatment enhances the ability of healthy cells to rescue light-damaged cells in our co-culture system, particularly by augmenting basal and maximal respiratory capacity.

In the human eye, the retinal pigment epithelial cells form a tight connection, establishing the outer retinal barrier^51^. ARPE19, a cell line of retinal pigment epithelial cells, exhibits a stable and well-defined cellular morphology, characterized by the orderly arrangement of actin filaments at the cell membrane and limited cell motility, thereby minimizing long-range interactions with neighboring cells. Our observations using live cell imaging revealed that treatment with Y-27632 induced notable alterations in the cytoskeletal organization of ARPE19 cells, including an increase in cell adhesion area, enhanced cellular contour, reduced density of actin filaments at the membrane, heightened cellular plasticity, a tendency towards extending dendritic structures for intercellular contact, and augmented cell motility. These cellular modifications induced by Y-27632 likely promote the formation of nanotubes among RPE cells.

Y-27632 has been shown to modify the subcellular localization of mitochondria, leading to their enhanced penetration into the cortical cytoskeleton. Additionally, Y-27632 induces the extension of mitochondria with dendritic-like structures, resembling mitochondrial axonal transport in neuronal cells. It has been suggested that inhibition of PI3K could result in the redistribution of energetically active mitochondria to the cortical cytoskeleton, thereby promoting the motility and invasion of tumor cells. This redistribution alters mitochondrial membrane dynamics and enhances random cell motility^43^. Studies have indicated that localized activation of energy sensors and the metabolic regulator AMPK can stimulate forward migration of mitochondria, maintain ATP content, and ensure the ATP supply required for cell migration^52^. These findings suggest that the redistribution of mitochondrial membranes induced by Y-27632 is a response to the energy requirements associated with changes in cytoskeletal structure, potentially facilitating mitochondrial transport across cells.

Mitochondria are highly active organelles that play a crucial role in facilitating exchange by modulating mitochondrial morphology^53^, ^54^. During the process of transfer, mitochondria must be detached from the donor cell's mitochondrial network and transferred to the recipient cell^6^, ^55^. Our findings indicate that Y-27632 enhances the number and length of individual mitochondria, thereby potentially improving mitochondrial transport and energy production.

Various proteins involved in mitochondrial transport have been identified, including Miro1, a GTPase located on the outer mitochondrial membrane that regulates mitochondrial movement and distribution by coordinating interactions with actin and microtubules^39^, ^45^, ^46^. Knockdown of Miro1 has been shown to impede microtubule-dependent mitochondrial movement, underscoring its importance in this process^46^. Furthermore, disruption of F-actin has been found to hinder mitochondrial penetration into the cortical cytoskeleton. It is plausible that Y-27632 promotes mitochondrial movement and penetration by enhancing mitochondrial membrane localization and stimulating microtubule polymerization. These findings unveil novel mechanisms through which Y-27632 modulates intercellular mitochondrial transfer and subcellular distribution.

Our data, along with existing research, provide support for the hypothesis that Y-27632 enhances the likelihood of tunneling nanotube (TNT) formation through the modulation of cytoskeletal dynamics, leading to an increase in microtubule-mediated mitochondrial transport. These alterations are implicated in the facilitation of TNT-mediated intercellular transfer of mitochondria. Future experiments are necessary to elucidate the specific molecular mechanisms underlying the regulatory effects of Y-27632 on the cytoskeleton.

## Conclusions

In summary, our study presents novel findings indicating that Y-27632 enhances the formation of tunneling nanotubes (TNTs) through its regulation of the cytoskeleton. Furthermore, we observed that Y-27632 influences mitochondrial distribution and transport by modulating microtubule polymerization and the mitochondrial transporter protein miro1. These results offer valuable insights for the advancement of TNT-mediated material exchange, particularly in the context of mitochondrial transfer, as a potential therapeutic strategy. By bolstering the endogenous mechanisms that facilitate TNT-mediated material transport, this approach holds promise for future therapeutic interventions.

## Methods

### Cell cultures

ARPE19 cells (ATCC, #CRL-2302) were cultured in DMEM/F12 (Gibco, #C11330500BT) medium supplemented with 10% fetal bovine serum (FBS) (VivaCell, #C04002-500) and 1% penicillin and streptomycin (Gibco, #15140-122). The cells were cultured in a humidified incubator at a temperature of 37°C with 5% CO_2_.

### Cell labeling

Cells were subjected to staining using Cell Trace Violet (Invitrogen, #C34557) in DPBS at a concentration of 10µM for 10 minutes at 37°C, followed by three washes with fresh medium.

### Construction of ARPE19 cell line with mitochondrial fluorescent labeling

ARPE19 cells were transduced with lentiviral vectors containing mitochondria tagged with GFP (Mito-COX8-GFP, SBI, #Cyto102-PA-1) or RFP (derived from Mito-COX8-GFP) to establish the ARPE19-mito-GFP and ARPE19-mito-RFP cell lines, as previously described^1^.

### ROCK inhibitor treatment

ARPE19 cells were exposed to varying concentrations of Y-27632 (Selleck, #S1049) including 0µM, 10µM, 20µM, 40µM, 100µM, 400µM, and 1000µM for a duration of 24 hours. Y-39983 (AbMole, #M5123, 10µM) and GSK269962A (Baiaolaibo, M02736, 10µM) were similarly used to treat ARPE19 cells for a duration of 24 hours.

### Cell co-culture

ARPE19-mito-GFP cells were seeded at a density of 1×10^4^ cells in a single well of a 24-well plate and co-cultured with either ARPE19 or ARPE19-mito-RFP cells at a 1:1 ratio. The co-culture was then treated with 40µM Y27632 for a duration of 24 hours.

### Transwell co-culture

ARPE19-mito-GFP cells were seeded on the upper chamber, while ARPE19 cells were seeded on the lower chamber of a transwell system (Corning, #3470) with 0.4μm pore size inserts. Both cell types were seeded at a density of 1 × 10^4^ cells per chamber and were co-cultured for 24 hours with the treatment of 40µM Y-27632.

### Quantification of Y-NTs and mitochondrial transfer rate

The quantification of TNTs was conducted in over 10 fields randomly selected from each sample. The results are presented as the ratio of intercellular nanotubes (Y-NTs) relative to the total number of cells counted in each experimental condition. The presence of mitochondria from donor cells in recipient cells labeled with cell trace violet was used to determine the rate of mitochondrial transfer among cells.

### Cytoskeleton staining and confocal microscopy

Cells were cultured in a 24-well plate with coverslips for 24 hours, followed by PBS washing and fixation in 4% paraformaldehyde for 30 minutes at room temperature. Actin filaments (F-actin) and microtubules were stained using Phalloidin (Thermo Fisher Scientific, #A22287) and anti-α-Tubulin antibody (1:500, Sigma, #T9026). Samples were observed using an Olympus microscope, with STED images were acquired using Abberior STEDYCON (Abberior Instruments GmbH, Göttingen, Germany) fluorescence microscope built on a motorized inverted microscope IX83 (Olympus UPlanXAPO 100x, NA1.45, Tokyo, Japan). Cell size and nanotube length were quantified using Olympus confocal software.

### Live cell imaging

Cover-glass chambers were used for culturing ARPE19 cells. After rinsing with warm PBS and changing to phenol red-free DMEM/F12 medium (Gibco, #11039021), live cell imaging was conducted using an Olympus microscope equipped with a 40X objective in a live cell imaging incubator set at 37°C with 5% CO_2_.

### Scanning electron microscope (SEM)

Cells (4×10^4^cells/well) were cultured in a 12-well plate with a coverslip for 24 hours. Subsequently, the samples were fixed with 2.5% glutaraldehyde (Solarbio, #P1126) for 30 minutes at room temperature and then stored at 4°C for over 8 hours. Following fixation, the samples underwent a series of washes with DPBS before dehydration. The dehydration protocol involved sequential immersion in 30%, 50%, 70%, 85%, 95%, and 100% ethanol for specific durations. Post-fixation and dehydration, the coverslips were air-dried and subjected to metal coating using an E-1045 ion sputtering apparatus. Imaging was performed using a HITACHI SU8010 microscope.

### Depolymerization of F-actin and microtubule

To assess the impact of F-actin and microtubules on the formation of nanotubes and mitochondrial transfer, cells were exposed to Cytochalasin B (MCE, #HY-16928, 10µM, 24 h) or Nocodazole (Sigma, #M1404, 50µM, 24h) to disturb F-actin or microtubule polymerization, respectively.

### Assessment of cell apoptosis

Apoptosis was assessed by the AnnexinV-FITC/PI apoptosis double staining kit (BD, #556547). Cells were harvested, washed, and stained with 5 μl of AnnexinV-FITC for 15 minutes, after which they were exposed to 5 μl of PI for 5 minutes. Flow cytometry was utilized for result analysis.

### Establishment of an *in vitro* mitochondrial injury model by light damage and co-culture system

ARPE19 cells were exposed to 19 klux blue light (330-340 nm) for varying durations (0, 0.5, 1, 1.5, 2, and 2.5 hours) to investigate mitochondrial dysfunction. A 2-hour exposure to blue light was chosen to induce a model of mitochondrial injury. Light-damaged and undamaged ARPE19 cells were co-cultured with healthy ARPE19 cells at a 1:1 ratio.

### Assessment of mitochondrial membrane potential

Mitochondrial membrane potential assay was performed using the MitoProbe™ JC-1 Assay Kit (Invitrogen, #M34152). Cells were treated with JC-1 staining solution for 20 minutes at 37°C, washed thrice with DPBS, and the fluorescence signal was analyzed via flow cytometry.

### Oxygen consumption rate (OCR)

The oxygen consumption rate (OCR) is a measure of the rate at which cells utilize oxygen. Mitochondrial function of ARPE19 cells was assessed using a seahorse XFp analyzer (Agilent Technologies). By sequentially administering drugs targeting the mitochondrial electron transport chain (ETC), key parameters indicative of mitochondrial function were obtained: (1) basal respiration, reflecting cellular energy demand in the resting state; (2) ATP production, determined by the reduction in oxygen consumption upon addition of 1.5 µM oligomycin, indicating mitochondrial ATP synthesis capacity; (3) Maximum mitochondrial respiratory capacity, calculated as the peak oxygen consumption following treatment with 0.5 µM uncoupling agent FCCP, representing the maximal respiratory rate achievable by the cells. (4) Non-mitochondrial respiration was assessed by inhibiting mitochondrial respiration with a combination of 2µM rotenone and antimycin A. (5) Spare respiratory capacity, defined as the difference between maximum and basal respiration, signifies the cell's ability to respond to increased energy demands.

### RT-qPCR

Total RNA extraction was performed using the Total RNA Extraction Kits (Fastagen, #220010). Reverse transcription was done using the HiScript III RT SuperMix for qPCR (+gDNA wiper) (Vazyme, #R323). Quantitative PCR was then performed using the AceQ qPCR SYBR Green Master Mix (Vazyme, #Q131). The primers used for miro1 as a reference gene were as follows: forward 5'-TGTTCAGCGAAAAACCTGAA-3', reverse 5'-TTCAGCATCATTGAGAGTACCA-3'.

### RNA-sequencing (RNA-seq)

All the RNA-seq samples were sequenced by the Illumina NovaSeq 6000 with read lengths of 150bp at opposite ends. All reads were mapped to Homo sapiens. GRCh38.94.chr with the default settings. Read counts were calculated using the FeatureCount. The cytoskeletal gene expression was calculated by the edgeR (|log2 KD/NC|>1, P < 0.05).

### Western blot

Cells were lysed in RIPA lysis buffer (Proteintech, #PR20001) supplemented with cOmplete, EDTA-free, EASY pack (Roche, 04693132001). The primary antibodies employed were anti-F-actin (1:500, Abcam, #ab205), anti-α-Tubulin (1:500, Sigma, #T9026), anti-Miro1 (1:500, Invitrogen, #PA5-42646), Goat anti-Mouse HRP (1:1000, Proteintech, #SA0001-1), and Goat anti-Rabbit HRP (1:1000, Proteintech, #SA0001-2).

### Morphometric analysis of the mitochondrial network

Mitochondrial morphology analysis and quantification were conducted as previously outlined^56^. Image preprocessing was carried out to enhance contrast, clarity, and background reduction for improved binarization. The binary image was then skeletonized using the 'skeletonize' function in ImageJ. The MINA macro extracted branch lengths and numbers from the Analyze Skeleton plugin output to compute parameters characterizing mitochondrial network morphology. Mitochondrial structures were categorized as "individuals" (punctate, rod-shaped, large/circular) or "network" (≥3 branches) (Figure S5C).

### Statistical analyses

Data were presented as mean±SEM or median ± interquartile rangeand analyzed with one- or two-way analysis of variance (ANOVA), Kruskal-Wallis test, Student's t-test or Mann-Whitney test using SPSS 25. A P-value of < 0.05 was considered as significant; ns, not significant.

## Supplementary Material

Supplementary figures and movie legends.

Supplementary movie 1.

Supplementary movie 2.

Supplementary movie 3.

Supplementary movie 4.

## Abbreviations

ROCK: Rho-associated kinase
TNT: Tunneling nanotubes
RPE: Retinal pigment epithelial
ARPE19: Adult Retinal Pigment Epithelial cell line-19
MSCs: Mesenchymal stem cells
GFP: Green fuorescent protein
CM: Cytoplasmic membrane
RFP: Red fuorescent protein
Y-NTs: Y-27632-induced nanotubes
Cyto B: Cytochalasin B
Noco: Nocodazole
DMSO: Dimethyl sulfoxide
