# Supplementary material for: ROCK inhibitor enhances mitochondrial transfer via tunneling nanotubes in retinal pigment epithelium
Source: Theranostics. 2024 Sep 9;14(15):5762–77. doi: 10.7150/thno.96508 (PMC11426248; doi:10.7150/thno.96508)
Supplement: Supplementary file 1 — Supplementary figures and movie legends. [file thnov14p5762s1.pdf]

Supplemental Figures

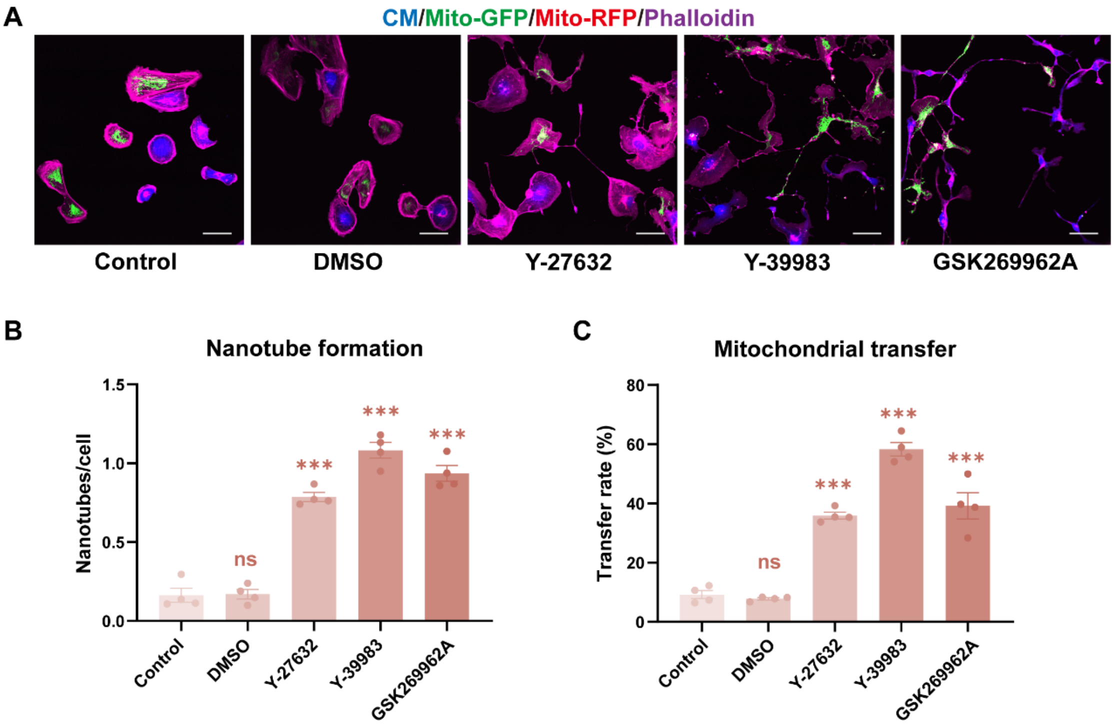

Supplemental Figure 1. ROCK inhibitors-induced intercellular nanotube formation and mitochondrial transfer.

(A) Representative images of ARPE19-mito-GFP (green) and ARPE19-mito-RFP cells (CM, blue; red) co-cultured and treated with different ROCK inhibitors for 24 hours. (B, C) The number of nanotubes each cell formed and mitochondrial transfer rate were quantified in control, DMSO, Y-27632 (40μM), Y-39983 (10μM) and GSK269962A (10μM) groups. n=8, one-way ANOVA test, mean±SEM; ns, not significant,***$p < 0.001$.

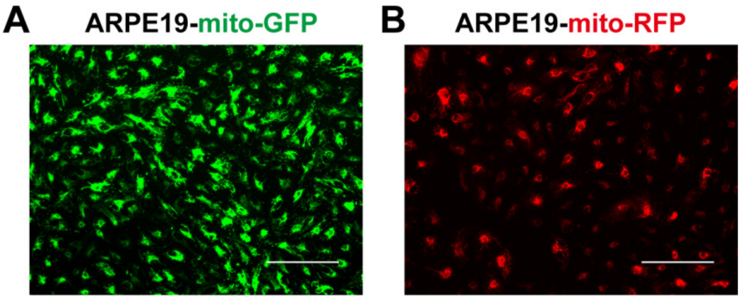

Supplemental Figure 2. Construction of mitochondrial fluorescently labeled ARPE19 cell lines

(A, B) ARPE19 cells were infected with lentivirus-containing mitochondria with GFP (mito-GFP) and RFP (mito-RFP).

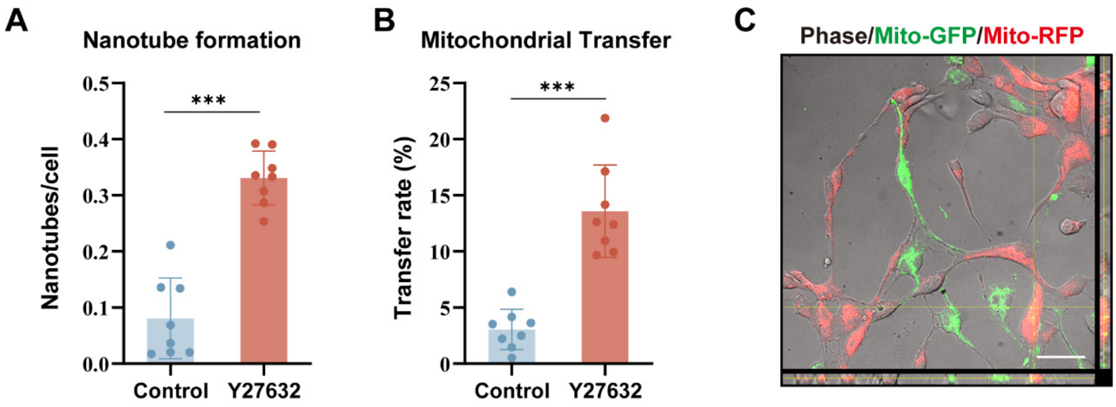

Supplemental Figure 3. Characterization of Y-27632-induced nanotubes

(A, B) The number of nanotubes each cell formed and mitochondrial transfer rate were quantified in control and Y-27632 (40μM for 24 h) groups. n=8, t test, mean±SEM; ***p < 0.001.

(C)Intercellular nanotubes curved with branching are formed between ARPE19-mito-GFP and ARPE19-mito-RFP cells following treatment with Y-27632. Scale bar: 50 μm.

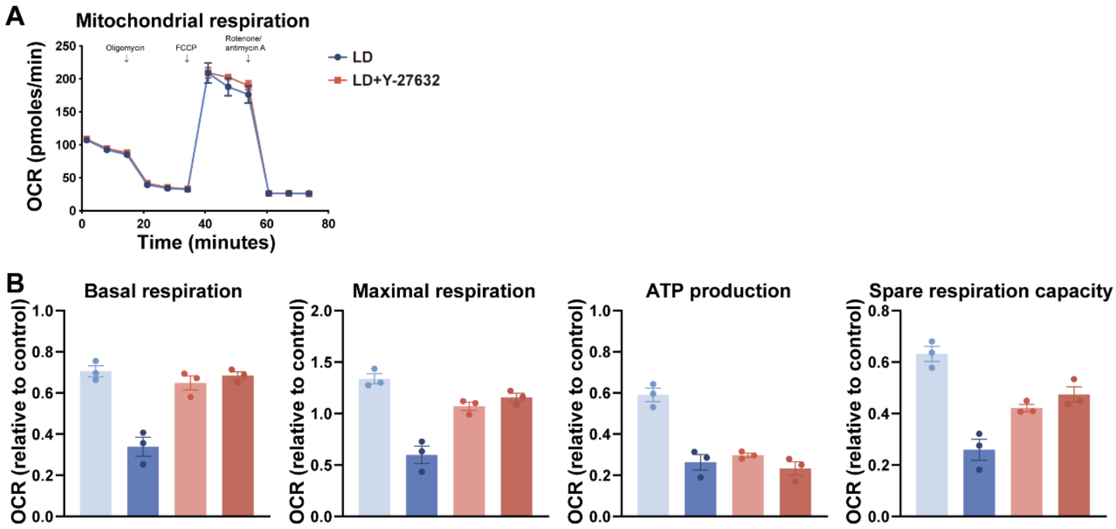

Supplemental Figure 4. Effect of Y-27632 on light-damaged ARPE19 cells

(A) Extracellular oxygen consumption rate (OCR) analysis of light-damaged ARPE19 cells with or without Y-27632 treated.

(B) Quantification of oxygen consumption rate (OCR) in mitochondrial basal respiration, maximal respiration, ATP production, and spare respiratory capacity.

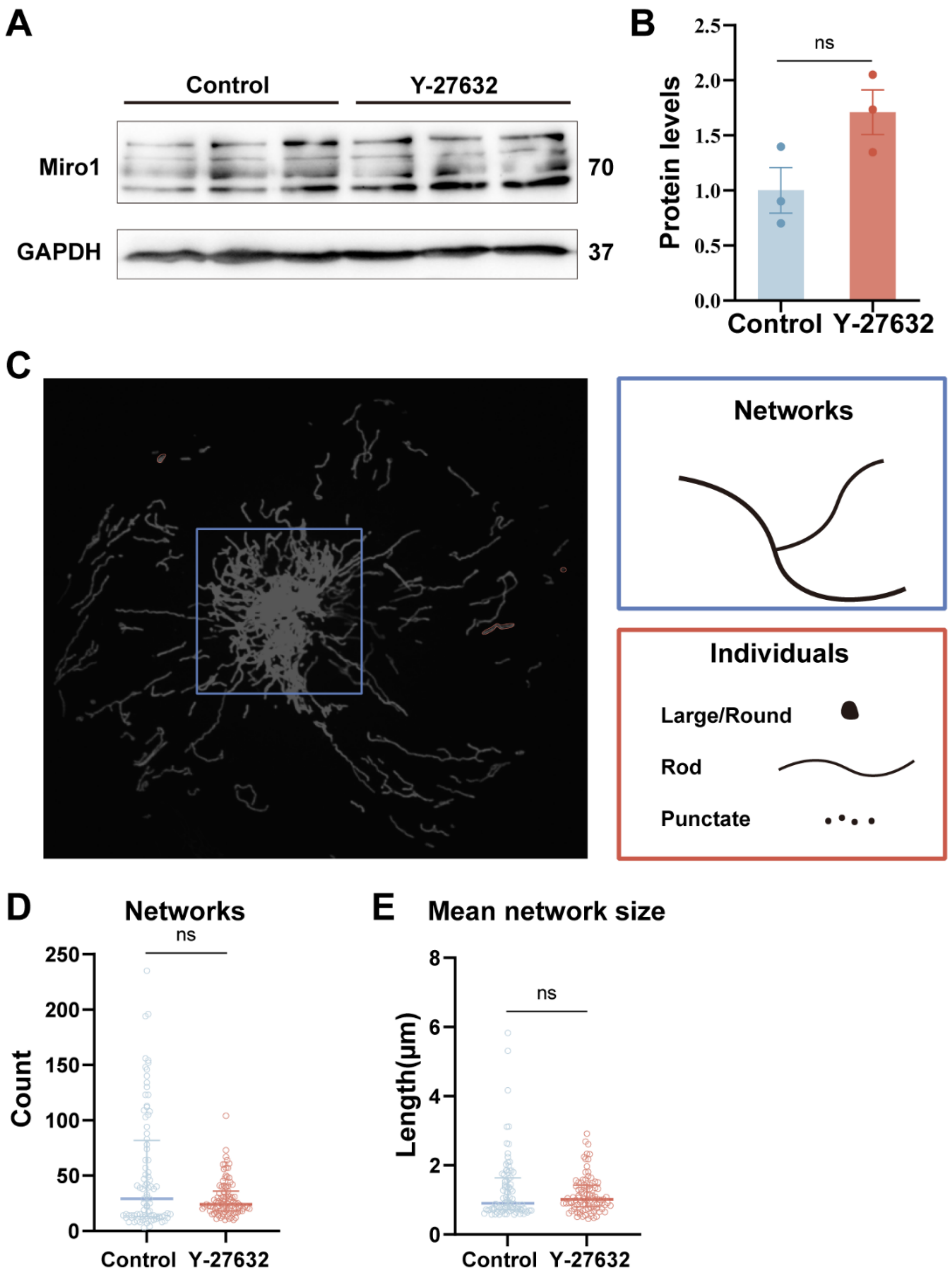

Supplemental Figure 5. Analysis of mitochondrial movement and network structure after Y-27632 treatment

(A, B) Miro1 protein levels were detected and quantified in control and Y-27632 groups by Western blotting. n=3, t test, mean±SEM; ns, not significant.

(C) Visual description of terms used in this study.

(D, E) The number and average size of mitochondrial networks in control and Y-27632 groups were quantified. n (control)=88, n (Y-27632) =95. Mann-Whitney test, median ± interquartile range; ns, not significant.

Movies

Movie 1. Y-NTs formation and mitochondrial transfer in bright field
ARPE19-mito-GFP cells (green) and ARPE19-mito-RFP cells (red, CM, blue) were co-cultured at a 1:1 ratio in direct contact.

Movie 2. Y-NTs formation and mitochondrial transfer with F-actin staining
ARPE19-mito-GFP cells (green) and ARPE19-mito-RFP cells (red, CM, blue) were co-cultured at a 1:1 ratio in direct contact, and all cells were stained with phalloidin (magenta).

Movie 3. Mitochondrial movement in ARPE19 cells with Y-27632 treatment
ARPE19-mito-GFP cells (green) and ARPE19 cells (CM, blue) were co-cultured at a 1:1 ratio in direct contact.

Movie 4. Mitochondrial movement in ARPE19 cells without Y-27632 treatment
ARPE19-mito-GFP cells (green) and ARPE19-mito-RFP cells (red, CM, blue) were co-cultured at a 1:1 ratio in direct contact.
